# Supplementary material for: Mitochondrial Dysfunction Underlies Cardiomyocyte Remodeling in Experimental and Clinical Atrial Fibrillation
Source: Cells. 2019 Oct 5;8(10):1202. doi: 10.3390/cells8101202 (PMC6829298; doi:10.3390/cells8101202)
Supplement: Supplementary file 1 [file cells-08-01202-s001.pdf]

Mitochondrial dysfunction underlies cardiomyocyte remodeling in experimental and clinical atrial fibrillation

Supplementary figures

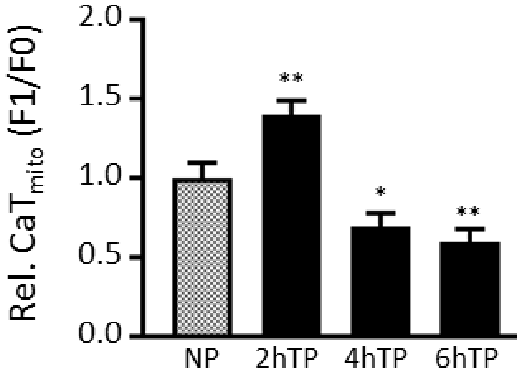

Figure S1 Buffering of Ca^{2+} by mitochondria. Mitochondrial calcium transient (CaT_{mito}) show an increase in mitochondrial Ca^{2+} amplitude upon initiation of tachypacing (TP) compared to normal-paced cardiomyocytes (NP), which gradually decreases upon longer periods of TP. *P<0.05, **P<0.01 vs NP.

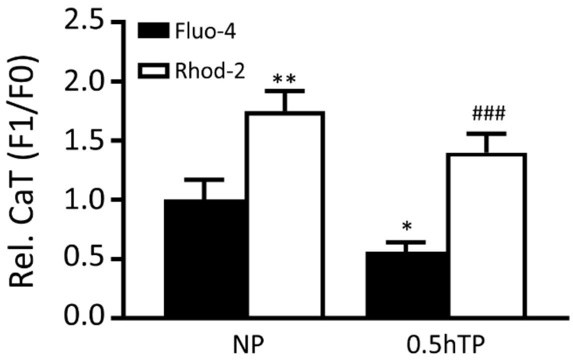

Figure S2 Rhod-2 measured CaT are an indication of changes in mitochondrial CaT. HL-1 cardiomyocytes simultaneously incubated with Fluo-4 (cytosolic CaT) and Rhod-2 (mitochondrial CaT) show Fluo-4 and Rhod-2 to be measuring cytosolic and mitochondrial CaT, respectively. After 0.5h of tachypacing (TP), cytosolic CaT are significantly decreased compared to normal-paced (NP), which is not the case for mitochondrial CaT. *P<0.05, **P<0.01 vs NP Fluo-4, ###P<0.001 vs 0.5hTP Fluo-4.

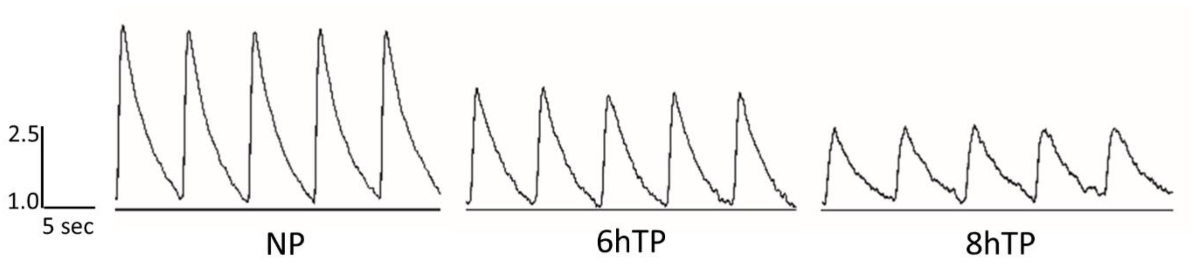

Figure S3 Tachypacing reduces CaT_{mito} amplitudes. Representative traces (5 seconds) of CaT_{mito} amplitudes of normal-paced (NP), 6h and 8h tachypaced (TP) HL-1 cardiomyocytes.

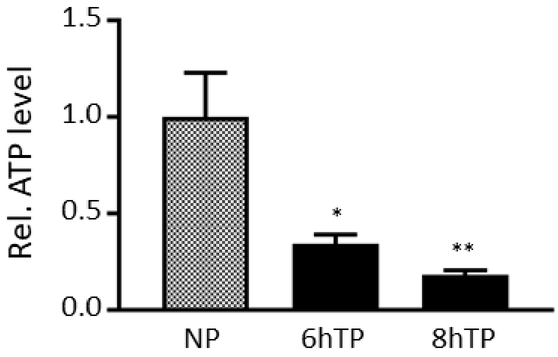

Figure S4 Tachypacing reduces cellular ATP levels. Cellular ATP levels are significantly decreased after 6hTP and 8hTP. **P*<0.05, ***P*<0.01 vs NP Fluo-4.

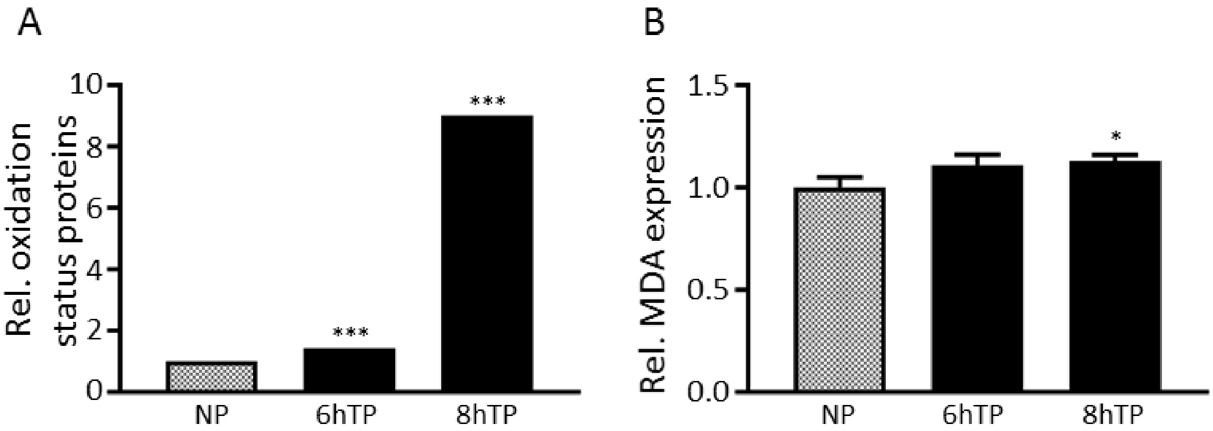

Figure S5 Tachypacing induces ROS. ROS levels are measured by **A)** oxidation status of proteins and **B)** MDA (malondialdehyde, lipid peroxidation) expression. In particular, ROS levels are increased after longer duration of tachypacing (8hTP) compared to normal-paced cardiomyocytes (NP). *P<0.05, ***P<0.001 vs NP.

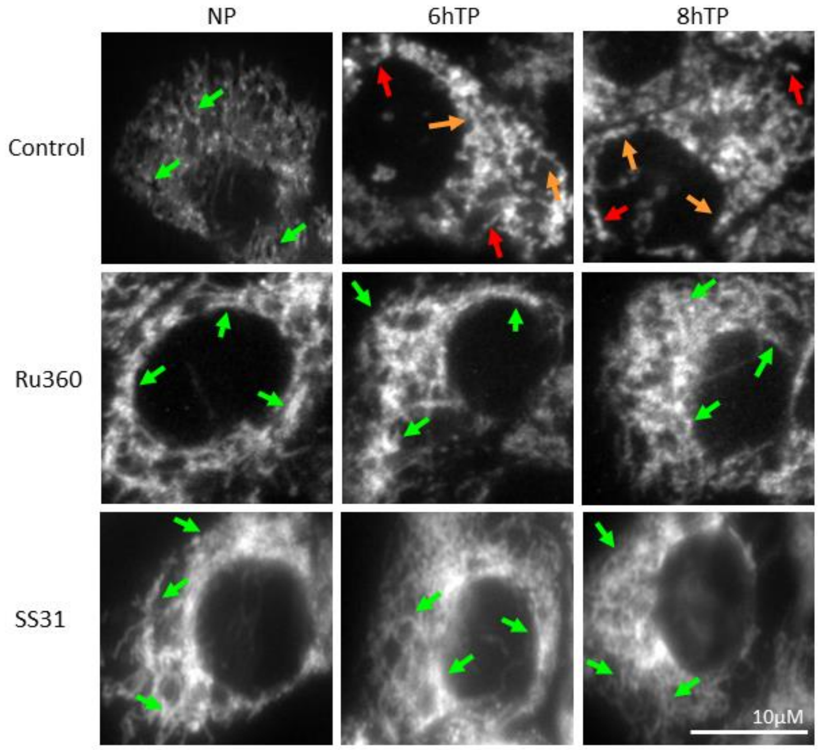

Figure S6 Changes in mitochondrial network morphology upon tachypacing and after treatment with Ru360 or SS31. Panels show representative confocal images of normal-paced (NP) and tachypaced (TP) HL-1 cardiomyocytes. NP cardiomyocytes show a tubular network, as depicted by the presence of long, intertwining tubules. After 6 and 8 hours of tachypacing (TP), the network is fragmented, as shown by the presence of single mitochondria (dots) and shorter tubules. Treatment with Ru360 or SS31 preserves the tubular network upon tachypacing. Green, orange and red arrows depict the tubular, intermediate and fragmented mitochondria,

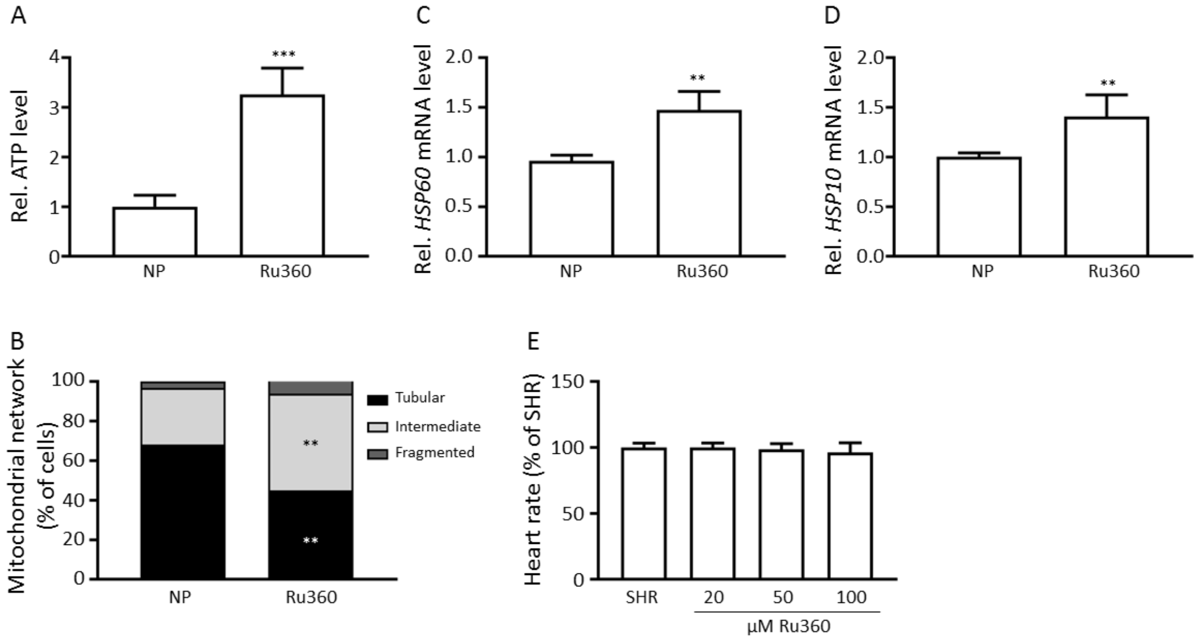

respectively.

Figure S7 The effects of Ru360 in normal-paced HL-1 cardiomyocytes. Ru360 treatment **A)** increases cellular ATP levels, **B)** changes the mitochondrial morphology and increases **C)** *HSP60* and **D)** *HSP10* mRNA levels of cardiomyocytes subjected to normal-pacing. **E)** Quantified heart wall contractions (heart rate) of *Drosophila*

monitored before tachypacing (SHR, spontaneous heart rate) with demineralized water (SHR) or Ru360 pretreatment. ***P*<0.01, ****P*<0.001 vs NP.

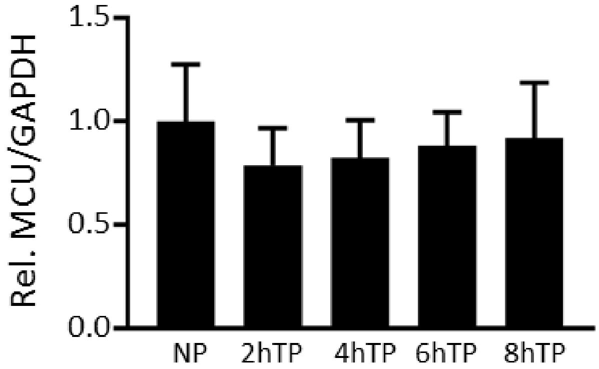

Figure S8 MCU expression during time course of tachypacing. Protein levels of MCU remains stable over a time course of tachypacing HL-1 atrial cardiomyocytes.

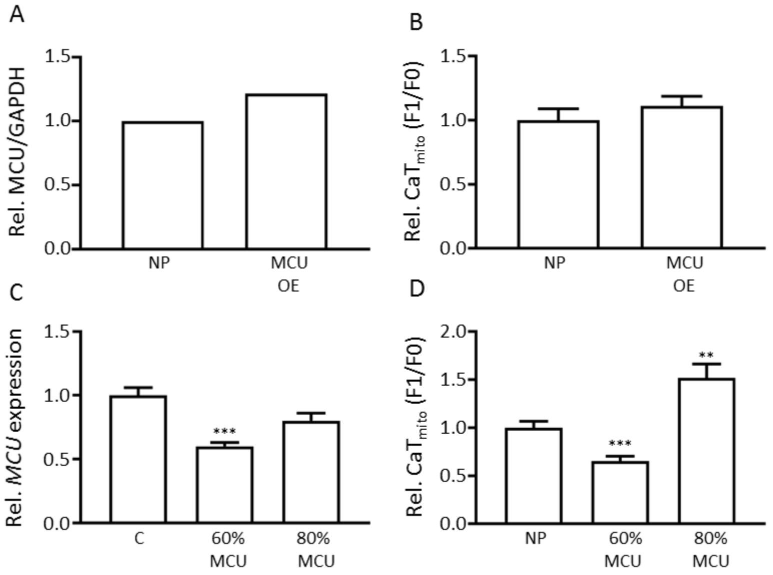

Figure S9 Overexpression of the MCU in normal-paced HL-1 cardiomyocytes does not change mitochondrial calcium transient amplitude, while downregulating the MCU does. A) Quantified data of MCU normalized for GAPDH, showing that transfection with MCU induces MCU overexpression on protein level. **B)** Quantified data of mitochondrial calcium transient (CaT_{mito}) amplitude of HL-1 atrial cardiomyocytes either non-transfected or transfected with MCU, generating MCU overexpression (MCU OE) subjected to normal-pacing (NP). **C)** Quantitative real-time PCR of MCU expression showing a 40% and 20% reduction of MCU mRNA compared to non-transfected cardiomyocytes (C). **D)** Quantified data of CaT_{mito} amplitudes of HL-1 atrial cardiomyocytes either non-transfected or transfected with MCU siRNA, generating MCU knockdown so that either 60% or 80% or MCU is still present after NP. ***P*<0.01, ****P*<0.001 vs NP/C.

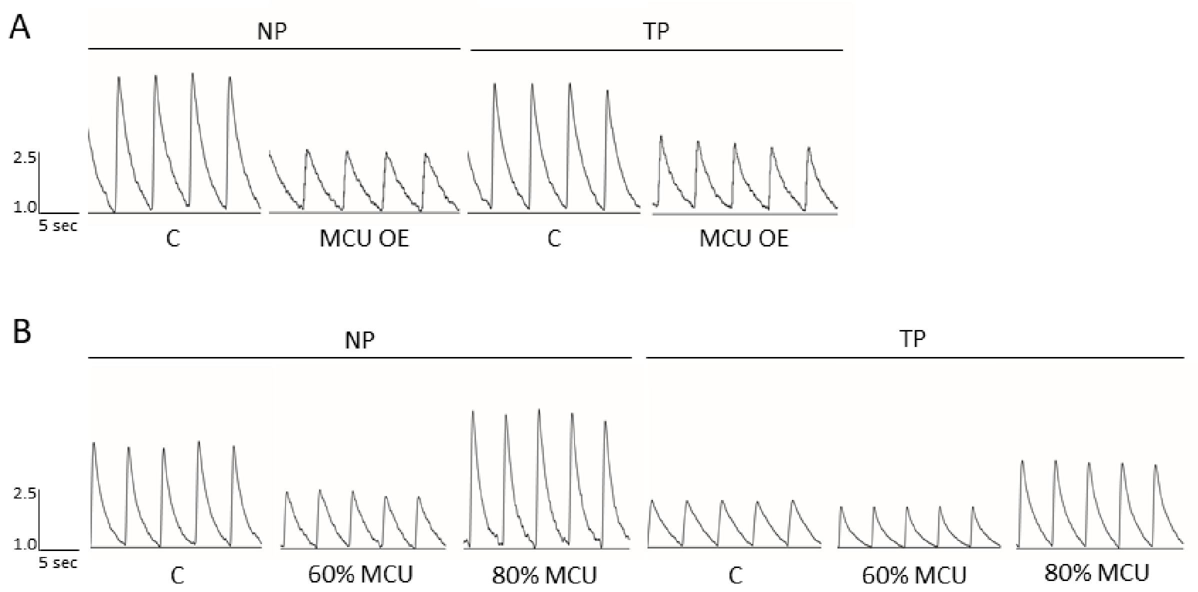

Figure S10 A modest reduction in MCU expression, but not overexpression, protects against tachypacing-induced CaT_{mito} amplitude loss. Representative traces (5 seconds) of CaT_{mito} amplitudes of normal-paced (NP) or tachypaced (TP) HL-1 cardiomyocytes with **A)** MCU overexpression (OE) or **B)** reduced MCU expression by siRNA treatment, resulting in 60% or 80% MCU expressed compared to control NP (C) in the cardiomyocytes.

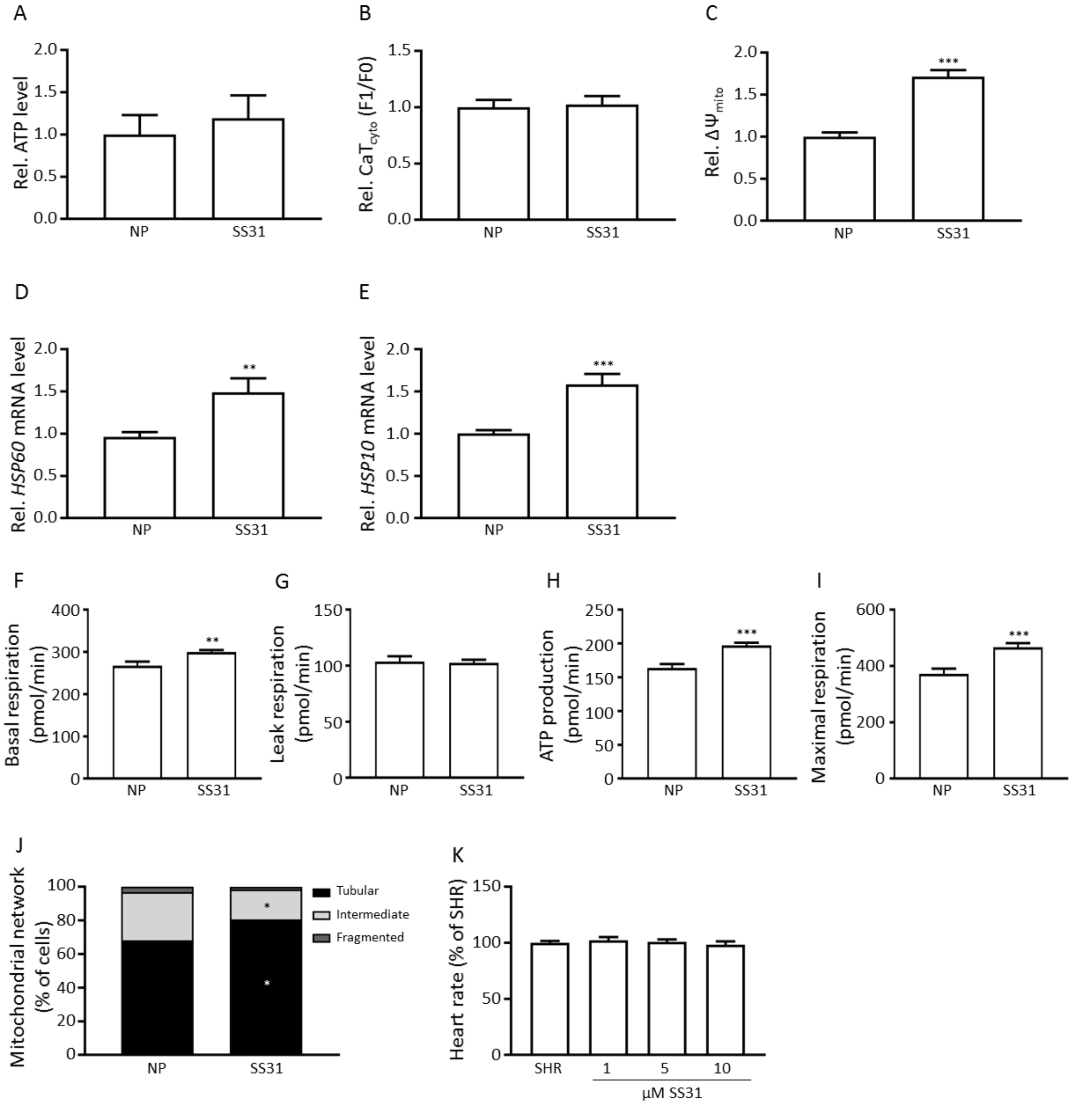

Figure S11 The effects of SS31 in normal-paced HL-1 cardiomyocytes. SS31 treatment does not change **A)** cellular ATP levels, **B)** cytosolic calcium transient (CaT_{cyto}) amplitudes and **G)** leak respiration. SS31 treatment induces **C)** the mitochondrial membrane potential ($\Delta\Psi_{mito}$), **D)** *HSP60* and **E)** *HSP10* mRNA levels, **F)** routine respiration, **H)** ATP production, **I)** maximal respiration and **J)** mitochondrial morphology (increased tubular network). **K)** Quantified heart wall contractions (heart rate) of *Drosophila* monitored before tachypacing (SHR, spontaneous heart rate) with demineralized water (SHR) or SS31 pretreatment. **P*<0.05, ***P*<0.01, ****P*<0.001 vs NP.

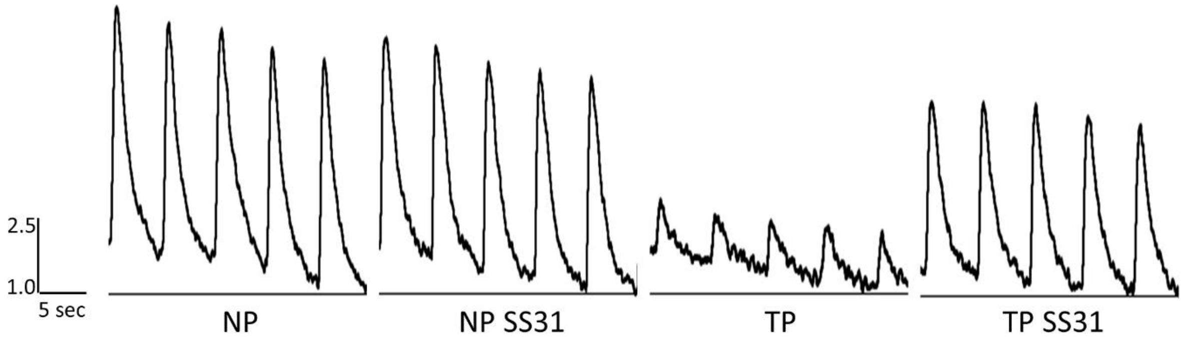

Figure S12 SS31 treatment partially attenuated contractile function. Representative traces (5 seconds) of cytosolic CaT amplitudes of normal-paced (NP) or tachypaced (TP) HL-1 cardiomyocytes without or with SS31 treatment.
